# In-Vivo and Ex-Vivo Measurements of Blood Glucose Using Whispering Gallery Modes

**DOI:** 10.3390/s20030830

**Published:** 2020-02-04

**Authors:** Louis W. Y. Liu, Abhishek Kandwal, A. Kogut, Z. E. Eremenko, E. Kogut, M. T. Islam, R. Dolia, S. Nosatiuk, Son T. Nguyen

**Affiliations:** 1 Faculty of Engineering, Vietnamese German University, Le Lai Street, Hoa Phu Ward, Thu Dau Mot City 75000, Vietnam; 2 SIAT Chinese Academy of Sciences, Shenzhen 518100, China; 3 O.Ya.Usikov Institute for Radiophysics and Electronics of NAS of Ukraine, 61072 Kharkov, Ukraine; kogut@ire.kharkov.ua (A.K.); rico911gt@gmail.com (R.D.);; 4 Institute of Radiophysics and Electronics, V.N.Karazin Kharkov National University, 61072 Kharkov, Ukraine; kogut@univer.kharkov.ua; 5 Department of Electrical, Electronic & Systems Engineering, Universiti Kebangsaan Malaysia, Bangi 43600, Malaysia; tariqul@ukm.edu.my; 6 Faculty of Engineering, Eastern International University, Binh Duong 75000, Vietnam; son.nguyen@eiu.edu.vn

**Keywords:** whispering gallery modes, blood glucose, non-invasive, fast wave, slow wave, surface wave, microwave, Goubau line

## Abstract

The permittivity of blood glucose is not a strong function of its concentration in microwave or millimeter-wave frequencies. Measuring glucose concentrations remains a challenge, particularly in the presence of interference caused by the ambient leaky waves. In this paper, however, we demonstrate that a near-linear correlation between the glucose concentration and the blood permittivity was noticeably observed at a whispering gallery mode resonance. Method: the proposed sensor was a vacuum suction aspirator partially wounded with a turn of the Goubau line. This arrangement enabled a fixed cylindrical volume of a skin tissue bump or glucose/water solution to be formed and used as a whispering gallery resonator for in-vivo and ex-vivo measurements. Results: in the in-vivo study, a near-linear correlation between the glucose levels and the S21 parameters was noticeably observed at the fundamental whispering gallery resonance (i.e., at 2.18 GHz). In the ex-vivo study, a similar correlation was observed between the concentration of a glucose/water solution and the S21 parameters 56.6 GHz. Conclusion: the results of both investigations were consistent not only with the invasive measurements using the Accu-check^TM^, but also with the conclusion drawn by some other research groups who have successfully measured blood glucose concentrations at millimeter-wave frequencies.

## 1. Introduction

In this paper, we present a sensor for non-invasive measurement of glucose concentration in both humans and phantoms without any chemical reagent. Measurement using electromagnetic waves is by far one of the most researched techniques because of its feasibility in continuous non-invasive blood glucose monitoring. However, the results of our investigation reveal that in-vivo or in-vitro measurement of glucose concentrations using electromagnetic waves is unlikely reliable unless the following issues are properly addressed:

(1) In microwave and millimeter-wave frequencies, as suggested by the Cole–Cole/Debye model [1], the permittivity of glucose/water solution is a weak function of glucose concentration. The permittivity changes are often too small to be observable even though there is a large swing in the glucose concentration within the range between 85 mg/dL and 500 mg/dL. In humans, normal blood glucose levels can be anywhere between 50 mg/dL and 120 mg/dL. Within the normal range, which is relatively small, the permittivity change due to a concentration swing can be too small to be detectable.

(2) The geometrical differences of the object to be measured from one experiment to another can critically affect the accuracy and reproducibility of the measurement at microwave or millimeter-wave frequencies. In order for a blood glucose measurement to be reproducible, the volume of the blood has to be the same in all experiments.

(3) In microwave and millimeter-wave frequencies, the glucose sensor can act like a leaky wave antenna, which is highly susceptible to ambient electromagnetic interference. If the leaky waves in the surrounding environment are not suppressed, a glucose sensor may be measuring ambient interference rather than the actual blood glucose concentration. Due to the presence of this interference, the measurement displayed by a sensor is potentially unstable and non-reproducible from one experiment to another. Whilst many studies have consistently shown promising results for measuring the dielectric properties of the biological tissues using different techniques [2,3,4], the problem of the leaky waves leading to non-reproducible measurements is rarely investigated.

As will be explained in the next section, however, the dependency between the glucose concentration and the permittivity becomes enormously amplified and increasingly observable when the frequency approaches a whispering gallery mode (WGM) resonance. This fact has been used to our advantage to resolve issue #1. To overcome issue #2, a vacuum suction aspirator was used. As will be explained in the next section, this vacuum suction aspirator can be cost-effectively used to form a fixed cylindrical volume either out of real human skin tissue or out of a glucose/water solution. To overcome issue #3, our glucose measurement was mainly based on whispering gallery modes. Electromagnetic waves can be broadly divided into two kinds: leaky waves and surface waves. In theory, leaky waves radiate to all directions whilst surface waves mainly propagate along the conducting or semi-conducting surface of the transmission media. Whispering gallery mode is one of the surface electromagnetic waves bound to the interior concave surface within a waveguiding structure. This mode is generated as a result of internal reflection by the interior concave surface. The glucose sensor to be proposed in this work was designed to suppress leaky modes and foster whispering gallery modes. Glucose concentrations were determined at frequencies where the whispering gallery modes resonate.

The feasibility of the proposed methodology has been experimentally proven to some extent in a study focusing on in-vivo blood glucose measurement at microwave frequencies [5]. However, in [5], the choice of frequency for determining the glucose levels has not been quantitatively explained. The analysis in [5] was based almost entirely on approximated Goubau line formulae, which has ignored the effect of thickness of the cylindrical volume of the skin tissue under the vacuum suction effect. In this work, instead of relying on the approximated Goubau line formulae only, we focus on the use of whispering gallery modes to investigate the measured results from a different perspective.

## 2. Materials and Methods

**Figure 1:**
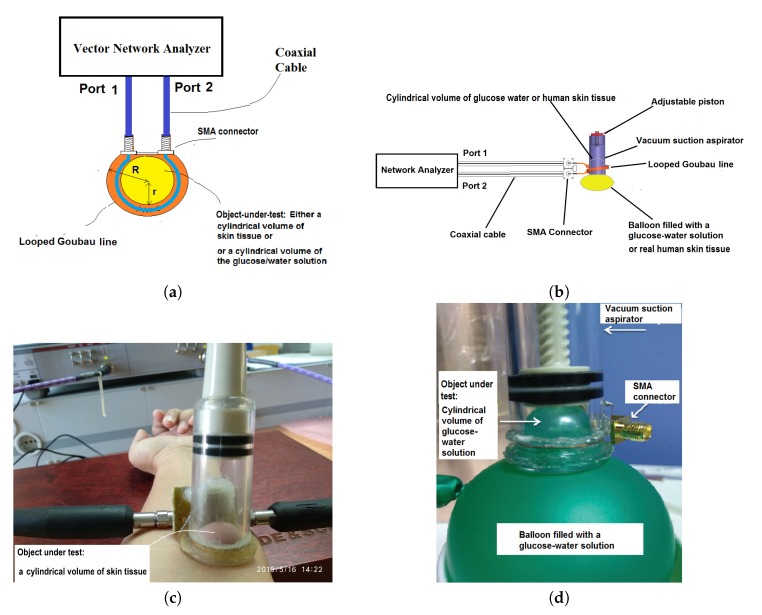
(**a**) Top view of the experimental setup for measuring the glucose concentration; (**b**) side view of the experimental setup for measuring the glucose concentration; (**c**) the proposed glucose sensor in an in-vivo experiment; (**d**) the proposed glucose sensor in an ex-vivo experiment.

Figure 1a shows the top view of the experimental setup for this work. In Figure 1a, the sensing head is the looped Goubau line, which is a dielectrically coated single-conductor transmission line. The object under test is either a cylindrical volume of a glucose/water solution in the case of an ex-vivo experiment, or a cylindrical volume of human skin tissue in the case of an in-vivo experiment.

Due to the near-cylindrical geometry of the object under test, the object under test can be thought in this scenario as a disk dielectric resonator. The looped Goubau line supports propagation of a surface electromagnetic wave, which is most likely a transverse magnetic wave (i.e., a TM mode). When the frequency of this surface electromagnetic wave matches the natural resonance frequency of the disk dielectric resonator, resonance will occur and a standing wave will be formed along the looped Goubau line. When the glucose concentration in this cylindrical volume changes, the permittivity corresponding to this glucose concentration changes accordingly. This permittivity change will induce a change in the natural resonance frequency of the disk dielectric resonator.

The key to harnessing the dielectric information from a glucose water solution or human tissue is to form a fixed volume of cylindrical disk dielectric resonator out of these materials. One way to achieve this goal was to use a vacuum suction aspirator as shown in Figure 1c or Figure 1d. The object under test is assumed to be flexible. Just before the measurement, the object under test is sucked into the proposed glucose sensor, forming a dump-like bump as shown in Figure 1c or Figure 1d.

The circumference of this vacuum suction aspirator was partially wounded by the looped Goubau line. The exterior wall of the vacuum suction aspirator is made with a low-k material known as acrylic plastic, which is almost transparent in microwave frequencies. The thickness of the exterior wall of the vacuum suction aspirator has been thinned down to 0.5 mm, which means the physical separation between the looped Goubau line and the object under test is basically negligible. Such a thin wall enables the looped Goubau line to sense the permittivity change of a phantom or a human skin tissue as a result of a change in the glucose concentration. For the purpose of description, the looped Goubou line together with the vacuum suction aspirator as shown in Figure 1c or Figure 1d is referred to as the proposed glucose sensor.

The looped Goubau line serves two purposes. First, the looped Goubau line excites the disk dielectric resonator during the measurement. Secondly, it is used to couple the electromagnetic energy released from the whispering gallery modes back to the Goubau line. Goubau lines are known to support propagation of a complex mix of multi-order surface modes, including the fundamental whispering gallery modes and other higher-order modes. In the proposed glucose sensor, the dielectric layer coating the Goubau line determines the cut-off frequency of surface modes (i.e., the cutoff frequency of the *m*-th order transverse electric mode) in accordance to the Siart [6]:

(1) $$f_{m,TE}=\frac{0.5cm}{t{\sqrt{\mu }}_{0}}{\left(\epsilon_{r}-\epsilon_{0}\right)}^{-1/2},$$

where the *c* is the speed of light. *t* is the thickness of the material coating the core metal in the Goubau line, *m* is the mode order, $\mu_{0}$ and $\epsilon_{0}$ are respectively the relative permeability and the relative permittivity of free space. $\epsilon_{r}$ is the relative permittivity of the coating layer of the Goubau line. Likewise, according to Siart [6], the cut-off frequency of the *m*-th order transverse magnetic mode can be approximated as:

(2) $$f_{m,TM}=\frac{0.5c(m+0.5)}{t{\sqrt{\mu }}_{0}}{\left(\epsilon_{r}-\epsilon_{0}\right)}^{-1/2}.$$

The diameter of the copper in the Goubau-line is about 1 mm. The thickness of the enamel coating of the Goubau-line is only 0.3 mm. The dielectric coating of the looped Goubau line is a mixture of gelatin and glycerol. By altering the proportion of these two chemicals, the equivalent relative permittivity and the conductivity of this dielectric coating were carefully adjusted to 44 and 6 S/m respectively. A surface mode along the looped Goubau line turns into a leaky wave at frequencies higher than its respective resonance frequency, or so-called cutoff frequency as defined by Equation (2). Below the cut-off frequency as defined by Equation (2), however, the electromagnetic energy will either exist as a whispering gallery mode along the looped Goubau-line or be absorbed into volume wave within the object under test (or the disk dielectric resonator). Neither the whispering gallery modes nor the volume waves are radiating.

In this work, the proposed glucose sensor as shown in Figure 1c,d has been used to conduct in-vivo or ex-vivo blood glucose measurements. In the case of the in-vivo experiment, the measurements were conducted on the surface of a human arm.

The in-vivo experiment was conducted in Vietnamese German University by a team of 30 healthy volunteers with no history of major illness. The measurements of S-parameters were conducted in a manner as shown in Figure 1c. In this case, the dielectric coating of the Goubou line winding the circumference of the glucose sensor is a 3 mm thick layer of solidified gelatin/glycerol composite, of which the permittivity and conductivity were determined to be 48 and 0.001 respectively. We have obtained measured S-parameters for a number of different blood glucose levels. In each of the in-vivo measurements, the volunteer was first asked to go through an invasive measurement using Accu-chek^TM^ first. After obtaining the Accu-chek measurement, the volunteer was assisted to non-invasively measure his/her blood glucose levels using the proposed glucose sensor. In this publication, the results for the following blood glucose levels are disclosed: 75 mg/dL, 101 mg/dL, 112 mg/dL and 140 mg/dL.

In the ex-vivo experiment, millimeter-wave measurements were conducted on a phantom. The phantom was a balloon filled with the glucose/water solution (see Figure 1d). The S-parameters were measured for 5 different concentrations of glucose in the glucose water, namely 100 mg/dL, 200 mg/dL, 300 mg/dL, 400 mg/dL and 500 mg/dL. According to Equations (1) and (2), a higher cutoff frequency means that a thinner dielectric coating of the Goubau line is required. In this work, in order for the looped Goubau line to operate at frequencies up to 67 GHz, we have chosen a layer of enamel of 0.3 mm thickness as the dielectric coating of the looped Goubau line (see Figure 1d).

All subjects gave their informed consent for inclusion before they participated in the study. The study was conducted in accordance with the Declaration of Helsinki, and the protocol was approved by the Ethics Committee of Vietnamese German University (VGU-EEIT-410).

## 3. Simulated Whispering Gallery Modes

The tissue bump or the balloon bump formed under the vacuum suction using the proposed glucose sensor is assumed to be near cylindrical and is treated as a disk resonator for the purpose of analysis. Even with this assumption, the tissue bump or the balloon bump does not have any sharp edge or corner. The top part of the disk dielectric resonator is essentially dome-like. The absence of any sharp edge or corner is a perfect condition enabling internal reflection to propagate in the form of whispering gallery waves inside the disk dielectric resonator.

**Figure 2:**
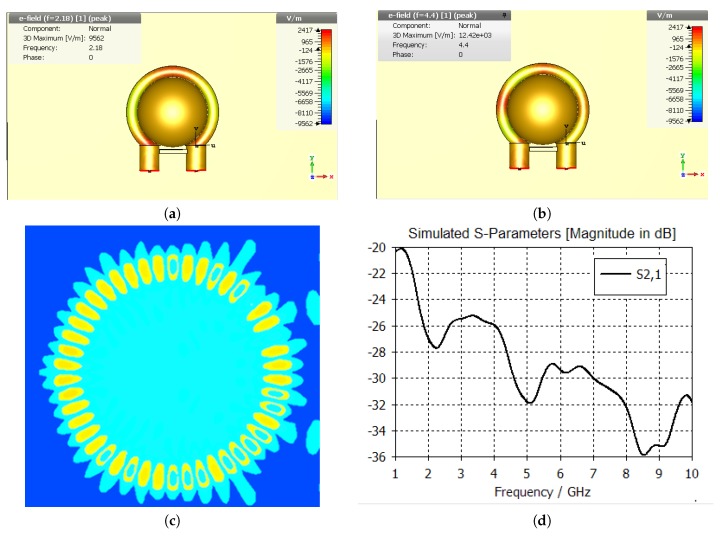
Simulated results (**a**) simulated whispering gallery resonance at 2.18 GHz, (**b**) simulated whispering gallery resonance at 4.4 GHz, (**c**) simulated whispering gallery modes at 56.2 GHz and (**d**) the simulated S-parameters for the proposed experimental setup.

To prove the existence of the whispering gallery modes, we have conducted a series of CST simulations according to the measurement setup as shown in Figure 1. The results of our CST simulation as shown in Figure 2 reveals that, with the help of the looped Goubau line, the circumference of the disk dielectric resonator yields a clearly defined whispering gallery resonance pattern in the neighborhood of certain frequencies, including 2.18 GHz, 4.5 GHz and 56.2 GHz. The three minima of the simulated S-parameters in Figure 2d is another proof suggesting the possible existence of whispering gallery resonance at 2.18 GHz and 4.5 GHz.

The electromagnetic characteristics of an object under test with whispering gallery modes can be deduced from the pattern of the simulated electric field. As shown in Figure 2a,b, the field distribution of whispering gallery modes inside the disk dielectric resonator can be obtained by counting the number of wavelengths along the circumference of the object under test. In this scenario, the number of red spots in Figure 2a suggests that the radial mode index is 1 at 2.18 GHz, i.e., m=1. On the other hand, there are 4 red spots in Figure 2b, suggesting that the radial mode index is 2 (i.e., m=2), at 4.4 GHz.

## 4. Justification of Measuring Glucose Concentration at Whispering Gallery Mode Resonance

The electromagnetic energy inside the object under test is a mixture of different hybrid modes. Hereafter, the object under test is referred to as a disk dielectric resonator. The hybrid modes in the disk dielectric include $EH_{nml}$ and $HE_{nml}$. These hybrid modes can be excited in the disk dielectric resonator through the use of the looped Goubau line as illustrated in Figure 1a. When the frequency of a traveling wave along the looped Goubau line matches the natural frequency of a whispering gallery modes in the disk dielectric resonator, a standing wave will be created in the form of whispering gallery modes. Three modal indexes *n*, *m* and *l* determine the number of resonant field variation along the azimuthal, radial and axial coordinates, respectively. The behavior of the electromagnetic field inside the resonator is given in cylindrical coordinates by an expression of the form:

(3) $$J_{n}\left(\beta \rho \right)\left\{\begin{array}{c} cos(hz)\\ sin(hz) \end{array}\right\}exp\left(i\left(n\phi +\omega t\right)\right),$$

where $J_{n}\left(\beta \rho \right)$ is the Bessel function of the first kind, β and *h* are the transverse and axial propagation constants, respectively, related by the equation [7]:

(4) $$\beta^{2}=k^{2}-h^{2},$$

where $k=\omega /c(\sqrt{(}\epsilon \mu ))$, c is the speed of light in vacuum and ω the angular frequency of the radiation; ϵ and μ are the real parts of permittivity and permeability of the material forming the resonator. In the following derivation, the real part of permeability will be assumed equal to 1.

The disk resonator is equivalent to an infinite waveguide with each axis characterized by an axial propagation constant, according to [7,8]. As to the whispering gallery modes in this work, an infinite circular dielectric waveguide is assumed, and special attention will be paid to the case of weak axial propagation constants (h<<k): a basic role will then be assigned to the modal index *n*.

Equation (4) is the most basic equation for calculating the spectral characteristics for the electrical and geometric parameters. In this case, the axial propagation constant is dependent on the height H of the disk dielectric resonator:

(5) h=p(l+1)/H.

The coefficient of axial propagation γ is close to 1 and becomes equal to 1 when the field tends to zero on the plane surfaces of the resonator, as in the case of a metal cavity [7]. Thus, expression (2) takes the form:

(6) $$\beta =\sqrt{k^{2}-h^{2}}=\sqrt{\epsilon \frac{\omega^{2}}{c^{2}}-{\left(\gamma \frac{\pi (l+1)}{H}\right)}^{2}}\approx \sqrt{\epsilon \frac{\omega^{2}}{c^{2}}-{\left(\gamma \frac{\pi (l+1)}{H}\right)}^{2}}.$$

Equivalently, Equation (6) can be rewritten as:

(7) $$H=\frac{\pi (l+1)}{\sqrt{\epsilon \frac{\omega^{2}}{c^{2}}-\beta^{2}}}.$$

In the disk dielectric resonator, when β=0, the volume waves in the disk dielectric resonator are converted into whispering gallery waves:

(8) $$\omega =\frac{c\pi (l+1)}{\sqrt{\epsilon }H}.$$

The cutoff frequency *f* for mode (l+1) is, according to Equation (8):

(9) $$f=\frac{c(l+1)}{2\sqrt{\epsilon }H}.$$

Below the cutoff frequency as defined in Equation (10), mode (l+1) dissipates as a dielectric loss into the volume waves. At frequencies above the cutoff frequency as defined in Equation (10), mode (l+1) radiates out as a leaky wave.

Differentiating Equation (10) with respect to ϵ yields an expression defining how much change in the cut-off frequency in response to the change in the permittivity:

(10) $$\frac{df}{d\epsilon }=-\frac{c(l+1)}{2H\epsilon^{3/2}}.$$

Blood glucose of glucose in the glucose/water solution is a lossy dielectric, meaning that ϵ is complex. At the non-resonant frequencies, the object under test (i.e., the disk dielectric resonator) will absorb the electromagnetic energy in the form of volume waves which would otherwise be lost in the form of leaky waves. Due to the presence of this volume waves, there will be dielectric loss in the form of heat at frequencies where there is no whispering gallery resonance but this dielectric loss is not susceptible to environmental electromagnetic interference. At the resonant frequencies, however, all the volume waves will be transformed into whispering gallery modes of which an integer number of wavelengths fits into the circumference of the disk dielectric resonator.

Equation (10) clearly shows that the mode index *l* has a multiplication effect. In response to a change in the glucose concentration in the skin tissue or in the phantom, a change in the permittivity of the solution will result in a larger shift in the cut-off frequency if the mode index *l* increases. On the other hands, as the mode index increases, the low-order leaky modes increasingly dominate the propagating modes. In the S-parameter measurement using a network analyzer, this shift in the cut-off frequency is often accompanied with a corresponding magnitude change in the attenuation (or S21) of the blood glucose in the following manner, according to Equation (10):

(11) $$\frac{d(S21)}{d\epsilon }=\frac{df}{d\epsilon }\cdot \frac{d(S21)}{df}=-\frac{c(l+1)}{2H\epsilon^{3/2}}\cdot \frac{d(S21)}{df}.$$

In Equation (11), the $\frac{d(S21)}{df}$ is the slope of the S21 in the S21 versus frequency plot. Equation (11) suggests that the glucose-concentration induced change in the S21 parameters increases as the slope of the S21 against frequency goes steeper. The slope of the S21 against frequency is normally steeper at frequencies very close to a whispering gallery resonant frequency. In other words, the glucose-concentration induced change in the S21 parameters will be maximum when the frequency is close to a whispering gallery mode resonant frequency. During the measurement processing, the glucose concentration should be monitored right at frequencies close to a whispering gallery mode resonant frequency.

The height of the disk dielectric resonator *H* is assumed to be the height of the tissue bump formed by the vacuum suction of the glucose sensor. By our estimation, *H* is approximately half of the diameter of the disk dielectric resonator, that is, H=9 mm. By substitution of the equivalent height H=9 mm and the blood permittivity ϵ=63 into Equation (7), the fundamental whispering gallery resonance is determined to occur at 2.18 GHz, which obviously agrees with the simulation results in Figure 2. The simulated resonances at around 4.7 GHz and 56.2 GHz as shown in Figure 2 respectively correspond to the whispering gallery resonances at the 2nd mode and the 26th mode, i.e., l=2 and l=26.

## 5. Results of In-Vivo Experiments

**Figure 3:**
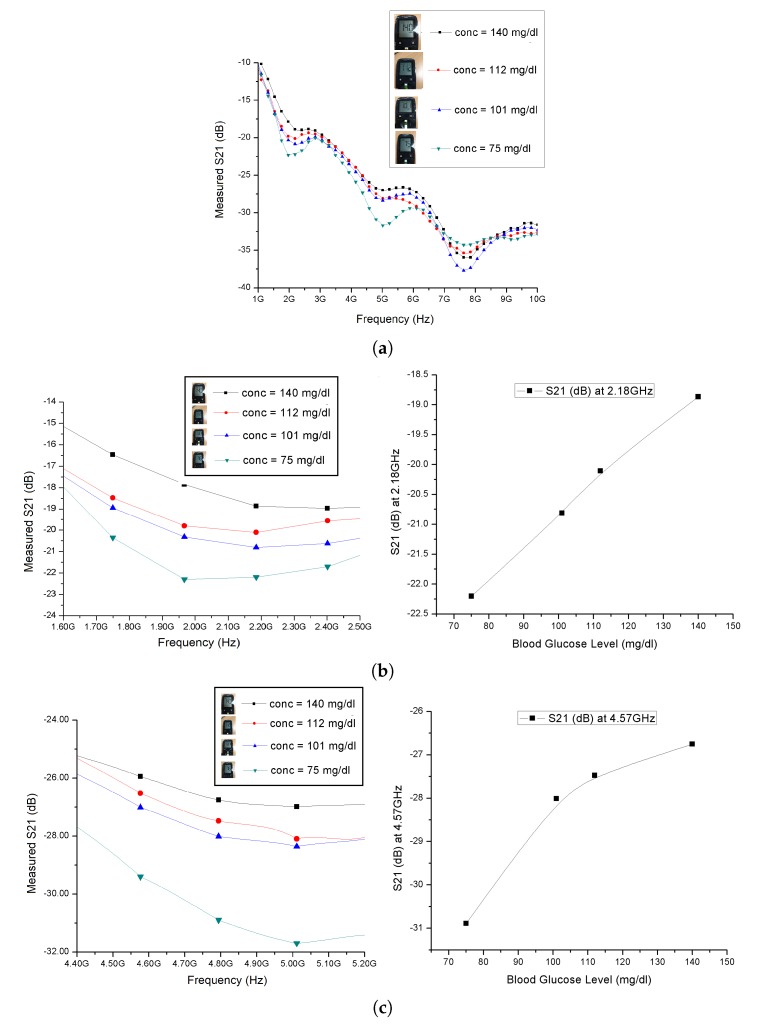
(**a**) Measured S21 from 1 GHz to 10 GHz; (**b**) measured S21 parameters at the 1st resonance (**left**), and the measured S21 as a function of blood glucose levels at the 1st resonance (**right**); (**c**) measured S21 at the 2nd resonance (**left**), and measured S21 as a function of blood glucose levels at the 2nd resonance (**right**).

Figure 3 shows the results of the in-vivo measurements conducted at the Vietnamese German University. Each of the measurements was individually conducted by one of the volunteers. For each of the measurements, the volunteer was assisted to take an invasive measurement using Accu-chek^TM^. This invasive measurement was immediately followed by the non-invasive measurement using the proposed glucose sensor. There was virtually no time gap between the invasive and non-invasive measurement.

As suggested in the measured S-parameters in Figure 3a, a mode resonance has occurred at 2.1 GHz, 5 GHz as well as 7.5 GHz. The measured S-parameters closely agree with the simulated S-parameters as shown in Figure 2d at least in terms of the resonant frequencies, confirming that the mode resonances found in the measured S-parameters were indeed whispering gallery mode resonances as expected by simulation.

As shown in Figure 3b,c, both the fundamental and second whispering gallery resonances have yielded a positive correlation between the S-parameters and the blood glucose levels obtained using a Accu-chek^TM^ meter. At the fundamental whispering gallery resonance, the correlation between the S-parameters and the blood glucose levels obtained by invasive measurement was nearly linear. The S21 parameter has noticeably changed over the whole measurement frequency range, particularly when the glucose levels fell between 77 mg/dL and 100 mg/dL.

All the in-vivo measurements have been found to be reproducible without any significant discrepancies.

## 6. Results of Ex-Vivo Experiments

In the ex-vivo experiment, we have prepared six phantoms for the ex-vivo measurements. Each of the phantoms is a balloon filled a glucose/water solution of a known glucose concentration. How the glucose-water filled balloon was sucked into the proposed glucose sensor has been described in Section 2. The following concentrations of glucose have been measured: 100 mg/dL, 200 mg/dL, 300 mg/dL 400 mg/dL and 500 mg/dL. The results have been reproduced in different experiments.

**Figure 4:**
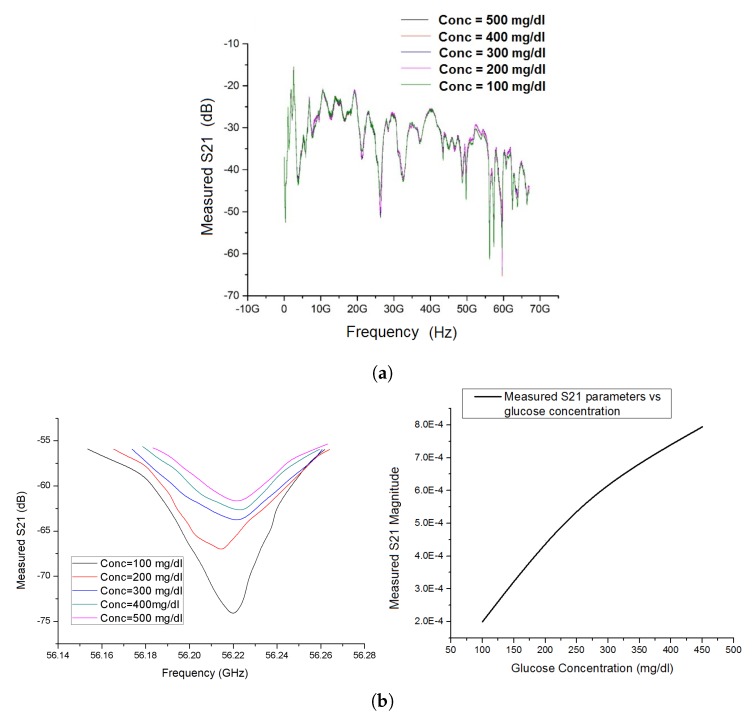
S-parameters measured using Keysight Signal Analyzer N9030A: (**a**) measured S21 from 1 GHz to 67 GHz; (**b**) measured S21 parameters focused in the neighborhood of 56 GHz (**left**), and measured S21 parameters as a function of glucose concentration at 56.2 (**right**).

Figure 4a shows the S-parameters over the frequencies from 1 kHz to 67 GHz for different concentrations of glucose. Upon careful examination, we found that 56.2 GHz was one of the few millimeter-wave frequencies in which a near-linear correlation has been established between the blood glucose concentrations and the S21 parameters. In Figure 4b, the diagram of the left shows a significantly enlarged plot of S21 parameter versus frequency with a focus on frequencies in the neighborhood of 56.2 GHz. As shown in the diagram on the right of Figure 4b, there exists a near-linear positive correlation between the S21 parameter and the blood glucose concentration at 56.2 GHz. The S21 parameter was found to be most sensitive to the blood glucose change at 100 mg/dL, which was within the normal blood glucose concentration of a healthy human.

## 7. Discussion

The results of our repeated measurements suggest that both the ex-vivo and in-vivo measurements yield a reproducible positive correlation between the measured S21 parameters and the glucose concentrations at the fundamental whispering gallery resonance, the second whispering gallery resonance and the resonance at 56.2 GHz. The results of our experiments and CST simulations consistently agree with each other.

The results of our in-vivo measurements were consistent with the results of the invasive measurements using Accu-Chek^TM^. Based on the measured S-parameters as shown in Figure 3a, the whispering galle10ry resonance frequencies closely agree with the values obtained using Equation (10). The finding of our in-vivo investigation has proven beyond any doubt that the frequency range from 1.8 GHz to 5 GHz can be used for in-vivo measurements.

As shown in Figure 4b, 56.2 GHz was one of the resonant frequencies yielding a near-linear correlation between the S21 parameters and the glucose concentration. It should be pointed out that the magnitude of the measured S21 parameters at 56.2 GHz was so low to the extent that it almost reached the noise floor of the network analyzer. Such a large attenuation was due to the low-order leaky modes being the dominant propagating modes. However, the results of our ex-vivo measurements consistently agree with the conclusion drawn by some other research groups who have successfully measured glucose concentrations at around the frequency range from 55 GHz to 65 GHz [9,10,11].

On the other hand, we failed to observe any positive correlation between the blood glucose levels and the S21 parameters at the third whispering gallery mode resonance and some other resonance frequencies, where the leaky modes were believed to be the dominant propagation modes.

The results of this investigation, though preliminary, at least prove the fact that the suction aspirator allows a fixed cylindrical volume of skin tissue or phantom to be measured using the set-up as shown in Figure 1a with reproducible glucose measurements at whispering gallery mode resonances. The milestone success in this work is still in its infancy. More research will be required before the clinical use of electromagnetic waves for monitoring blood glucose levels can become a reality.

## 8. Conclusions

In this work, the glucose sensor has been successfully formed by a vacuum suction aspirator in conjunction with a looped Goubau line. In-vivo study: with the help of this glucose sensor, we have conducted an in-vivo clinical trial on a team of 30 volunteers at the Vietnamese German University. In each in-vivo measurement, the volunteer was asked to do both the invasive measurement using Accu-check^TM^ and the non-invasive measurement using the proposed glucose sensor. A correlation was drawn between the glucose levels measured by the invasive method and the S21 parameters measured by non-invasive measurements. Ex-vivo study: separately, an ex-vivo experiment has been conducted on a glucose/water solution of the following concentrations: 100 mg/dL, 200 mg/dL, 300 mg/dL, 450 mg/dL and 500 mg/dL. In both measurements, a fixed cylindrical volume was formed out of a real human skin tissue in the case of in-vivo measurement, or out of a blood/glucose solution in the case of ex-vivo measurement. Whispering gallery mode resonances were extracted and used for determining the in-vivo and ex-vivo blood glucose measurements. Results: the results of the in-vivo study suggest that the fundamental whispering gallery resonance has occurred at 2.18 GHz and that there exists a positive correlation between the measured S21 parameters and the human glucose levels at the first and second whispering gallery resonant frequencies. The results of our ex-vivo study suggest that at 56.2 GHz, there exists a near-linear relationship between the S21 parameters and the blood glucose concentration. The finding of our ex-vivo study agrees with the conclusion drawn by some other research groups who have experimentally confirmed the feasibility of measuring the blood glucose levels at frequencies around 60 GHz.

## Abbreviations

The following abbreviations are used in this manuscript:

MDPI	Multidisciplinary Digital Publishing Institute
DOAJ	Directory of open access journals
